# Dissociating Sensory and Cognitive Biases in Human Perceptual Decision-Making: A Re-evaluation of Evidence From Reference Repulsion

**DOI:** 10.3389/fnhum.2019.00409

**Published:** 2019-11-15

**Authors:** Shenbing Kuang

**Affiliations:** State Key Laboratory of Brain and Cognitive Science, Institute of Psychology, Chinese Academy of Sciences, Beijing, China

**Keywords:** reference repulsion, sensory bias, cognitive bias, motion perception, reference frame

## Abstract

Our perception of the world is governed by a combination of bottom-up sensory and top-down cognitive processes. This often begs the question whether a perceptual phenomenon originates from sensory or cognitive processes in the brain. For instance, reference repulsion, a compelling visual illusion in which the subjective estimates about the direction of a motion stimulus are biased away from a reference boundary, is previously thought to be originated at the sensory level. Recent studies, however, suggest that the misperception is not sensory in nature but rather reflects post-perceptual cognitive biases. Here I challenge the post-perceptual interpretations on both empirical and conceptual grounds. I argue that these new findings are not incompatible with the sensory account and can be more parsimoniously explained as reflecting the consequences of motion representations in different reference frames. Finally, I will propose one concrete experiment with testable predictions to shed more insights on the sensory vs. cognitive nature of this visual illusion.

## Introduction

A long-standing question in perception and cognitive science is to address how our perception of the world is driven by a division of labor between bottom-up sensory processes and top-down cognitive factors. When a misperception happens, we cannot help but asking, is the perceptual illusion caused by bottom-up sensory mechanisms, or is it due to top-down cognitive processes? This dissociation is crucial, because the stage at which the visual illusion arises has important implications for the underlying neural mechanisms. Narrowing down the sources of visual illusions is therefore a promising and popular approach for better understanding the function and organization of human perceptual and cognitive systems.

Perception of motion is one important aspect of human vision, and one compelling visual illusion during motion perception is the so-called reference repulsion – a systematic bias away from a static reference line when estimating the direction of a cloud of moving random-dot stimuli (Rauber and Treue, 1998). Systematic biases in motion perception have also been identified between two spatially superimposed moving stimuli (also known as transparent motion) (Marshak and Sekuler, 1979). While motion misperception in the latter is widely considered to be a sensory process – arising from inhibitory interactions between neighboring neurons in motion sensitive cortical areas encoding these two motion directions (Mather and Moulden, 1980; Hiris and Blake, 1996; Kim and Wilson, 1996; Deng et al., 2017), the mechanisms mediating reference repulsion are still in great controversy (Rauber and Treue, 1998; Wiese and Wenderoth, 2008). It is previously suggested that reference repulsion is sensory in nature, reflecting the consequence of the decoding strategy optimized for direction discrimination around the reference boundary (Jazayeri and Movshon, 2007). This sensory view, however, is contradicted by recent findings arguing that reference repulsion is not a sensory process but rather reflects late, post-perceptual cognitive biases (Zamboni et al., 2016; Fritsche and de Lange, 2019).

This paper intends to re-evaluate these two contradictory accounts of reference repulsion within the context of the existing literature. I will argue that the post-perceptual cognitive account is incompatible with well-documented empirical evidence that reference repulsion depends systematically on low-level sensory features. I will contend that these recent studies fall prey to conceptual and methodological shortcomings, which makes the post-perceptual interpretation questionable. I will show that these new findings can be fully explained as reflecting the consequence of visual motion representations in different reference frames – a view that is within the framework of the sensory account. In the end, I will suggest one concrete experiment with testable predictions to shed more insights on the dissociation of sensory vs. cognitive account for this visual illusion.

## The Sensory Vs. Post-Perceptual Cognitive Account

Studies on reference repulsion typically involve two successive phases: an early discrimination phase in which subjects view a motion stimulus and discriminate its direction against a reference boundary by pressing one of the two keys on a keyboard, and a subsequent estimation phase, in which they estimate the direction of motion by drawing a line from the center of the display with a computer mouse (Rauber and Treue, 1998). In a previous study, Jazayeri and Movshon (2007) found that reference repulsion occurred when the direction estimation was preceded by a fine direction discrimination task (judging whether the direction of motion was clock-wise or counter clock-wise relative to the reference line), but not when it was preceded by a coarse discrimination task (judging whether the direction of motion was toward or away from the reference line). With decisive insights gained from computational models, the authors convincingly showed that reference repulsion arises from the sensory decoding process – a bimodal weighting profile of sensory evidences employed by participants to optimize the fine direction discrimination performance around the reference boundary (Jazayeri and Movshon, 2007).

The recent study by Zamboni et al. (2016) tackled this issue from a different perspective. To disentangle the sensory vs. decision-related cognitive accounts, the authors manipulated the timing and orientation of the reference line during the estimation phase (while keep the early sensory stimulus unchanged) in two separate experiments. In one experiment, for half of the trials, subjects performed the fine discrimination task before direction estimation – the reference line was displayed throughout the trial, closely matching the procedures of the previous Jazayeri and Movshon (2007) study to replicate the findings. For the remaining trials, the reference line was removed during the estimation phase. Their logic was, if the misperception is a consequence of sensory mechanisms (which operates during the early discrimination phase), then the removal of the reference line during the estimation phase should not affect the phenomenon. Intriguingly, the authors found that reference repulsion was eliminated when the reference line was removed during the estimation phase [or greatly reduced with a more sensitive data analysis in a later study (Luu and Stocker, 2018)]. In addition to the manipulations on the timing of the reference line, in another experiment Zamboni et al. (2016) further manipulated the orientation of the reference line (unknown to subjects). It was shown that misperception of motion direction was anchored to the orientation of the reference line during the estimation phase, irrespective of its orientation during the preceding discrimination phase. With these evidences, the authors concluded that reference repulsion is not sensory in nature, i.e., motion representation in the brain is veridical. Instead, the misperception arises from late, post-perceptual decision biases during the estimation phase.

Very recently, Fritsche and de Lange conducted further interesting manipulations on the reference repulsion task to shed more insights on the issue (Fritsche and de Lange, 2019). They first replicated the phenomenon that after discriminating the orientation of a grating stimulus against a reference boundary, the subsequent estimation response (adjusting the orientation of a bar) was repelled from the boundary, even though the reference boundary was presented prior to both the discrimination and estimation phases. In a second experiment, they employed a different protocol during the estimation phase. Instead of asking participants to reproduce the perceived orientation of the grating stimulus with bar adjustments, the authors measured their perceived orientation more directly, in comparison to a new reference boundary simultaneously presented on the opposite side of the preceding discrimination stimulus. Their results revealed that participants exhibited only a small repulsive bias that was indistinguishable from random fluctuations of sensory representations. The absence of systematic bias in this more direct, perceptual measurement led the authors to conclude that reference repulsion does not reflect sensory biases during the early perceptual stage, but rather stems from late, post-perceptual decision or working memory related biases.

## Limitations of the Post-Perceptual Interpretations

In my opinion, both Zamboni et al. (2016) and Fritsche and de Lange (2019) studies are important and intriguing new findings regarding the nature of reference repulsion. Yet, I do not fully agree with their post-perceptual interpretations on these new data. I will argue that the post-perceptual interpretations are at odds with the fact that the degrees of repulsion are sensory property dependent, e.g., larger repulsion occurs when the direction of motion is closer to the reference boundary and when the motion stimulus contains higher level of noise (low coherence). I will then challenge their interpretations on both conceptual and methodological grounds. I will show that these new data can be fully accounted for within the context of the previous sensory view proposed by Jazayeri and Movshon (2007).

First, the post-perceptual account of reference repulsion is hard to reconcile with the widely replicated empirical evidence that the magnitude of repulsion is dependent on the sensory properties of the motion stimulus. For example, repulsive biases are larger when the angular distances between the direction of motion and the reference boundary are small (Rauber and Treue, 1998; Rauber and Treue, 1999; Grunewald, 2004; Jazayeri and Movshon, 2007; Luu and Stocker, 2018). The degrees of repulsion are also sensitive to the coherence level of the motion stimulus: the biases become larger when the motion stimuli are less coherent (more noisy) (Jazayeri and Movshon, 2007; Luu and Stocker, 2018). These sensory property dependence of reference repulsion has been well characterized not only in previous studies but also in these two recent studies under discussion (Zamboni et al., 2016; Fritsche and de Lange, 2019). So, if reference repulsion arises at the later, post-perceptual stages, as suggested by the authors, repulsive effects should be insensitive to these manipulations on the early visual features (which presumably exert more influence over perception than cognition). The fact that reference repulsion depends systematically on both the coherence and the direction of motion indicates that sensory mechanisms contribute to the occurrence of repulsion and that the repulsive bias is introduced, at least partially, during early visual processing in striate or extra-striate visual cortex where cells are sensitive to low level sensory features (Gros et al., 1998).

Second, on the conceptual level, a bias probed during the late post-perceptual stage does not unequivocally prove that the bias arises at the late stage. In visually guided behaviors, since visual processing is the basis for all subsequent processes (e.g., memorizing, decision-making), a bias introduced early during visual processing affects not only visual perception *per se* but also all subsequent processing stages. To put it more specifically, let’s assume that reference repulsion is a perceptual bias. Images of a repulsed direction of motion will be perceived and stored in visual short-term memory of participants. Direction estimation based on these biased perception and memory will lead to persistent deviations even if no extra biases are introduced at late decisional stage. Therefore, the processing stage at which the repulsion is read out does not necessarily signal its origins. Nevertheless, since sensory processing is at the very forefront of these processing stages (an arguably privileged position), the fact that variations in sensory processing modulate the magnitude of repulsion suggests that it is likely a perceptual bias. But one cannot argue in a similar fashion for the post-perceptual account. When the repulsion is sensitive to post-perceptual manipulations, it is more complex: the bias could occur potentially at any node along the information flow ranging from the early sensory processing to late memory and decision stages. In this sense, it is bold to state that reference repulsion is not a perceptual bias, without thoroughly considering and refuting the sensory account.

Third, the two recent studies fall prey to methodological limitations which make their post-perceptual interpretations less credible. Importantly, I will show in the following section that the new data presented in these two studies could be fully explained within the framework of the sensory account of reference repulsion, without resorting to the post-perceptual arguments which contain more provocative assumptions and are less straightforward.

## An Alternative Sensory Explanation for These New Findings

On one hand, the manipulations on the reference line (or the reference boundary) in these two recent studies have introduced new frames of reference in which the visual information are represented, and these differences in the spatial representations can fully explain their data (i.e., the presence vs. absence of repulsion observed in various experimental conditions). The idea of visual representation in different reference frames is not an new concept in perception (Wade and Swanston, 1996). We know that an external motion or orientation can be encoded relative to the direction of gaze (retinotopic) (Colby and Goldberg, 1999), or to the body (spatiotopic) (Turi and Burr, 2012; Melcher and Morrone, 2015), or to another external object of relevance (object-based) (Agaoglu et al., 2015; Agaoglu et al., 2016). An example for object-based motion perception is that, we perceive the wheels of a passing car rotating while their actual retinal trajectories are cycloid (because wheel motion is coded relative to the car body). These different frames of reference for visual representation are selected flexibly by the perceptual system according to the task demand and the relevance of the behavioral context. For example, in the Zamboni et al. (2016), the authors manipulated the timing of the reference line visibility. When the reference line was visible during both the discrimination and estimation phases, the direction of motion was coded relative to the same object (the reference line) and reference repulsion occurred. However, when the reference line was only visible during the discrimination phase but not the estimation phase, the direction of motion was coded relative to the reference line for the discrimination task but perhaps to the more prevailing screen edges for the estimation task. In other words, the computations of motion signals for solving these two tasks are based on independent visual information (reference line vs screen edge). Consequently, the discrimination and estimation processes become unrelated so that the fine discrimination task will no longer exert its influence on subsequent estimation task. Therefore, misjudgments did not occur (Jazayeri and Movshon, 2007).

It is worthwhile to point out that when the two tasks are related (i.e., when reference repulsion occurs), there is an interesting behavioral performance trade-off: participants seem to sacrifice their estimation accuracy (biased away from the true direction) in order to achieve optimized fine direction discrimination performance. As a result, the estimation performance is surprisingly less accurate when a smaller, spatially focal line is chosen as the reference for motion computation, relative to bigger screen edges as the references. I speculate that motion representation relative to the bigger screen edge may be more accurate (since repulsion is absent) but less precise (i.e., large variability); in contrast, motion representation relative to the smaller reference line may be less accurate (since repulsion is present) but more precise. Whether this pattern of estimation accuracy vs. precision trade-off is valid needs to be tested with experimental data in future studies.

The same criticism of introducing different reference frames for motion processing applies to the Fritsche and de Lange (2019). In that study, participants were explicitly instructed to discriminate the orientation of a grating stimulus against one reference boundary presented on the left visual hemifield, and then estimate its orientation in comparison to another reference boundary presented on the right hemifield. It thus makes sense for participants to code the orientation of the grating stimulus relative to two different boundary stimuli. This may be the very trivial reason why the preceding orientation discrimination did not affect the subsequent orientation estimation in their data, because the computations for these two tasks are again based on two unrelated visual information represented in different spatial coordinates.

One might argue that the visual information might be transferred and shared across reference frames, so that to effectively observe a repulsive phenomenon, the stimuli in the discrimination and estimation tasks do not have to be represented in the same spatial coordinate. To test this idea more deeply, let’s momentarily hypothesize that reference repulsion is indeed a sensory bias. The sensory view predicts that the repulsion should be present when the direction is estimated by bar orientation adjustments, and be absent when the direction is estimated by direct comparison to a simultaneously presented reference stimulus at a different location because the repulsive effects will cancel out between the perception and estimation phases (since visual information at different locations are shared). To better understand this logic, I will take “wearing sunglasses makes the world appear darker” – a sure perceptual phenomenon – as an example: if we measure the influence of wearing sunglasses on the brightness of an object by means of grayscale rating, we will see an effect; however, if we measure it by asking participants to compare the brightness of that object to a another reference object, we will not see an effect because the effects of sunglasses on the two objects cancel out each other.

In general, a hypothesis or theory can be tested either in a confirmatory fashion – you should observe an effect when your hypothesis predicts its presence, or in a dis-confirmatory fashion – you should not observe an effect when your hypothesis predicts its absence. In the context of reference repulsion, the data presented by Fritsche and de Lange (2019) provide both confirmatory (their first experiment) and dis-confirmatory evidence (the second experiment) supporting the sensory account.

To briefly sum up, the idea of different reference frames elicited by the task designs in these two recent studies can fully account for their new data, no matter we consider visual processing strictly as a spatially specific process, or it is transferable across the visual field. Admittedly, different processing as a result of changes in reference frames is not necessarily indicative of perceptual mechanisms. For instance, in the famous Necker cube illusion, the three-dimensional cube appears to alternate between two different orientations, depending on which face the brain interpret it as being the front face of the cue. Apparently, the reverses in this bi-stable visual experience are not triggered by sensory processes (because the visual information stay unchanged), but are due to variations in the internal cognitive states of the observers (Long and Toppino, 2004; Kornmeier and Bach, 2012). However, distinct from these perceptual bi-stability phenomena, the visual input in the two recent reference repulsion experiments has big noticeable changes (e.g., the reference line was presented at different locations, or was made absent). It is hence more parsimonious to assume that changes of reference frames in these studies reflect differential perceptual processing (instead of cognition) which leads to different degrees of repulsive effects.

On the other hand, these two recent studies dissociated the perceptual vs. post-perceptual bias accounts using a temporal separation in their task designs which explicitly presumed that visual and cognitive processing are successive modular brain functions that can be strictly separated in time. While it is reasonable to serialize the visual and cognitive processing in the task design for the sake of simplifying the nature of our responses to external stimuli, the extent of this temporal separation at the conceptual level remains debatable. Contemporary theories of visual perception have rigorously challenged this modular view and the extent of the temporal division between perception and cognition (Vetter and Newen, 2014; Firestone and Scholl, 2015). Instead, it calls for a generous blurring of the boundary between visual processing and high-level cognitive processing (Goldstone et al., 2015). In these two studies, it is unlikely that the visual processing has strictly followed the temporal division as assumed by the authors. In contrast, since subjects know they need to perform a dual task in each trial (discrimination and estimation), the mental computations of these two tasks are probably planned and unfold simultaneously as soon as the relevant visual information becomes accessible. At one extreme, if the visual and cognitive processing are perfectly overlapped in the strictest sense, the time point at which the repulsion is probed (via task manipulations) provides no decisive hints on where it is originated. At the other extreme, if the visual and cognitive processing are temporally separated processes (e.g., first perception then cognition), as I have elaborated in the previous paragraph, a repulsion sensitive to early manipulations during visual processing indicates that it may arise at the perceptual stage; but you cannot argue similarly for the late post-perceptual stages. A repulsion sensitive to late post-perceptual manipulations does not unequivocally prove that it is a cognitive bias, since the observed repulsion could potentially be inherited from the early sensory processing stages. From this perspective, the temporal separation in the task designs of these two studies (with manipulations at the late stage) is probably not an ideal approach for decisively isolating the perceptual vs. post-perceptual biases.

## Future Experiments and Broader Perspectives

**FIGURE 1:**
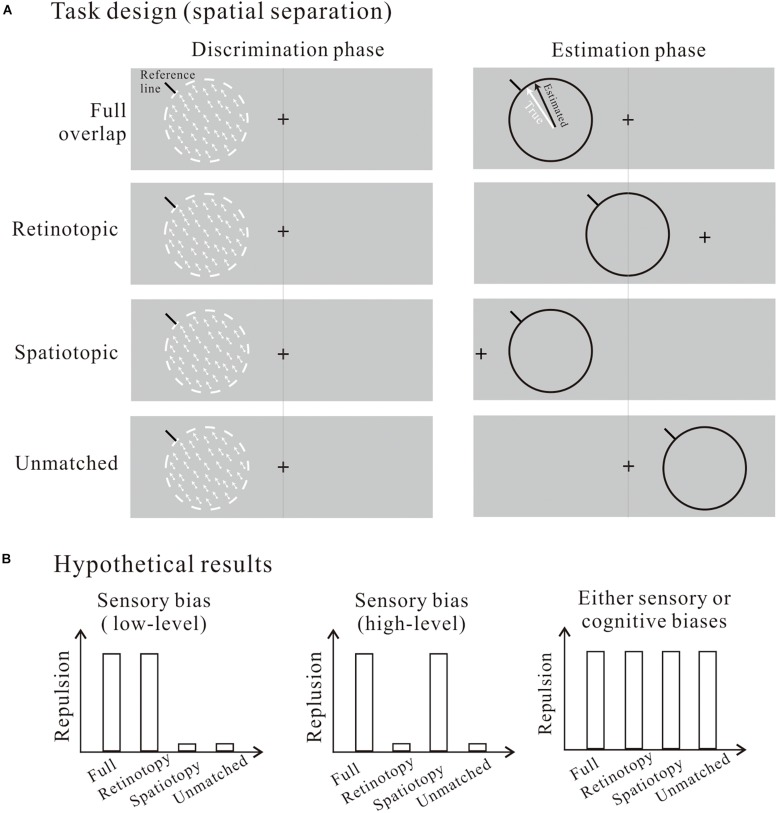
**(A)** Task design with spatial locations of the whole stimuli (the motion stimulus and the reference line) manipulated to be aligned retinotopically, spatiotopically, both, or neither between the discrimination and estimation phases. **(B)** Hypothetical results favoring either sensory (low-level and high-level) or post-perceptual cognitive biases as the sources of the repulsion.

Future studies with more carefully designed manipulations on the task parameters are needed to better disentangle the sensory vs. cognitive account of reference repulsion. As a starting endeavor in this direction, I here suggest one experimental design with different testable predictions to offer some new insights on this dissociation. Instead of manipulating the reference line alone, one could consider varying the spatial location of the entire stimuli (so the relative position between the motion stimulus and the reference line are unchanged between the discrimination and estimation phases). Combining this with appropriate gaze controls we can achieve the following four conditions (Figure 1A): (1) full overlap: the whole stimuli are at the same retinal and screen locations; (2) Retinotopic: they are at the same retinal location only; (3) Spatiotopic: they are at the same screen location only; and (4) Unmatched: they are spatially independent. Contrasting the repulsive effects across these conditions will provide important implications on where the repulsive bias may arise. The working hypothesis is, if reference repulsion is a retinotopically specific phenomenon, i.e., the repulsive bias is selectively present only when the discrimination and estimation stimuli have shared retinotopic positions (Figure 1B, left panel), this will imply that reference repulsion may be attributable to low-level sensory mechanisms in retinotopically organized, early visual areas (Wandell et al., 2007). In contrast, if reference repulsion is a spatiotopic phenomenon, i.e., the repulsion occurs only when the two stimuli have the same spatiotopic positions (Figure 1B, middle panel), this will suggest that reference repulsion is a high-level sensory effect in higher visual areas where spatioptic representations are most prevalent (Turi and Burr, 2012; Melcher and Morrone, 2015). The third possibility is that reference repulsion occurs in all conditions (Figure 1B, right panel), and this will provide no decisive hint on the sources of the bias. The sensory vs. cognitive account cannot be disambiguated in this scenario, because the repulsion could be due to either top-down cognitive biases that act globally and thus making the repulsive effects independent of visuospatial manipulations, or bottom-up sensory biases that are inherited by subsequent post-perceptual processing stages (e.g., memorizing, decision-making, etc.). In the latter case, although the initial sensory biases are retinotopically or spatiotopically organized, bias representations at subsequent cognitive stages are independent from these coordinates. For example, information storage in visual short-term memory and decision-making are both believed to involve prefrontal and parietal association areas where spatial representations are more abstract (Fedorenko et al., 2013; Serences, 2016). Therefore, the repulsion can be observed in all conditions independent of these spatial manipulations even if it is originating at the sensory levels. The advantages of this spatial separation task design are twofolds. First, it does not introduce differences in the frames of reference under which the motion information is represented for the discrimination and estimation tasks, because the relative positions are fixed across the two phases. Second, it allows us to test the perceptual vs. post-perceptual account without explicitly conceptualizing a temporal division of labor between the visual and cognitive processing.

More broadly, reference repulsion is a general visual phenomenon that is not restricted to motion processing (McKone et al., 2001; Sweeny et al., 2012). Our brain tends to deploy extra neural resources to sharpen perception around important category boundaries, and this comes with a seeming cost in the concomitant repulsive bias away from the boundary. It could be argued that such distortions are beneficial for the perceptual system because it reduces across-category misjudgments in the presence of sensory noise (i.e., achieving optimized categorization performance) (Kourtzi, 2010). Whether these repulsive effects are caused by distorted neural representations in early sensory areas or in downstream cognitive areas remains to be tested. Future studies with novel experimental designs and neurophysiologic recordings are needed to unambiguously disentangle the sensory vs. cognitive account of reference repulsion. One should bear in mind, though, that like in many other perceptual phenomena (Intaite et al., 2013), bottom-up sensory and top-down cognitive mechanisms are not necessarily mutually exclusive in reference repulsion. We might need a hybrid conceptualization of interacting sensory and cognitive biases that are coordinated and integrated in determining the degrees of repulsive effects in different behavioral contexts.

## Author Contributions

SK is the sole author of this manuscript. He conceived the study and wrote the manuscript.

## Conflict of Interest

The author declares that the research was conducted in the absence of any commercial or financial relationships that could be construed as a potential conflict of interest.
